# Impact of the COVID-19 Pandemic on Cancer Clinical Trials: Alliance for Clinical Trials in Oncology Experience (Alliance A152022)

**Published:** 2024-07-25

**Authors:** Rebecca A Snyder, Shauna L Hillman, Veronique Marcotte, Electra D Paskett, Suzanne George, Olwen Hahn, Sumithra J Mandrekar

**Affiliations:** 1 Department of Surgical Oncology, University of Texas MD Anderson Cancer Center, Houston, Texas, USA; 2 Department of Health Services Research, University of Texas MD Anderson Cancer Center, Houston, Texas, USA; 3 Department of Quantitative Health Sciences, Alliance Statistics and Data Management Center, Mayo Clinic, Rochester, Minnesota, USA; 4 Division of Cancer Prevention and Control, College of Medicine, The Ohio State University, Columbus, Ohio, USA; 5 Dana-Farber/Partners Cancer Care, Boston, Massachusetts, USA; 6 Alliance Protocol Operations Office, University of Chicago, Chicago, Illinois, USA

**Keywords:** COVID-19, Clinical trials, Telemedicine, Accrual trends, National Clinical Trials Network (NCTN), National Community Oncology Research Program (NCORP), Pandemic, Protocol deviations

## Abstract

**Objective:**

The COVID-19 pandemic led to immediate changes in cancer clinical trial conduct. The primary aims of this study were to summarize the impact of the pandemic on Alliance for Clinical Trials in Oncology (Alliance) enrollment, protocol deviations, COVID-19 events (positive or presumptive-positive COVID test), and premature study discontinuation rates.

**Methods:**

Enrollment trends were examined from January 2019 (pre COVID-19 pandemic) through 2022. Data were captured for protocol deviations and premature treatment and study discontinuation events across all Alliance protocols using a centralized Medidata Rave database, and summarized from January 1, 2020, through June 30, 2022. Descriptive statistics and graphical techniques are used to summarize observed trends.

**Results:**

Overall enrollment across Alliance trials decreased during the COVID-19 pandemic and remained below pre-pandemic levels in 2022. Racial and ethnic demographics of enrolled patients did not change substantially. 4805 protocol deviations were reported on 2745 unique patients, with at least one protocol deviation reported by 618 sites and 77 unique trials. Commonly reported deviations were telemedicine visits (n=2167, 45%) and late/missed study procedures (n=2150, 45%). A total of 826 COVID-19 events were reported in 659 unique patients. Of an estimated 18,000 enrolled patients, only 68 withdrew from treatment and 45 withdrew from study due to COVID-19.

**Conclusion:**

A centralized COVID-19 database enabled a comprehensive assessment of the impact of the pandemic across Alliance trials. COVID-19 led to an immediate decline in enrollment across all patient populations. While the number of trials open to patient accrual remained stable, several large, adjuvant studies completed accrual during this period, which contributed to accrual decline. Telemedicine usage was notable, and both COVID-19 events and study discontinuation due to COVID-19 were rare.

## INTRODUCTION

The COVID-19 pandemic in 2019 affected millions of Americans, including patients with cancer [1]. Early data suggested that patients with cancer or on anti-cancer therapy had a higher likelihood of severe COVID-19 infection and a higher COVID-19 fatality rate compared to the general population [2]. There were also major concerns that patients infected with COVID-19 would likely experience delays or disruptions in cancer therapy, which could result in worse long-term outcomes [3]. Additionally, the pandemic led to immediate changes in the conduct of cancer clinical trials through efforts to limit viral exposure for patients and research staff. Both the National Cancer Institute (NCI) and the U.S. Food and Drug Administration (FDA) released guidelines in March 2020 to address challenges faced by clinical trial sites and to provide guidance to aid clinical researchers and cancer clinical trial sites in mitigating immediate hazards to patients during the pandemic [3–5]. Recommendations included allowing remote consenting for enrollment, allowing patients to be treated by local physicians instead of at the trial center, mailing oral drugs to patients (*versus* requiring in-person dispensing), and conducting clinical and safety assessments *via* telemedicine.

To understand the impact of COVID-19 on the treatment and outcomes of oncology patients, several registry studies led by the NCI and by the American Society of Clinical Oncology (ASCO) were initiated [6,7]. These efforts sought to describe the clinical course of infection among oncology patients testing positive for COVID-19 and modifications to cancer therapy and cancer outcomes by clinicopathologic characteristics. However, these studies did not explore the effect of the pandemic on NCI-sponsored clinical trial enrollment or trial procedures.

The purpose of this study was to evaluate the impact of the COVID-19 pandemic on the conduct of Alliance for Clinical Trials in Oncology (Alliance) National Clinical Trials Network (NCTN) and National Community Oncology Research Program (NCORP) studies. Our primary aim was to understand and summarize clinical trial enrollment trends over time and by patient demographic characteristics. We sought to examine these data for all patients enrolled on Alliance clinical trials, regardless of COVID-19 status. Our secondary aim was to summarize the impact of COVID-19 on protocol deviations and premature treatment and trial discontinuation rates.

## METHODOLOGY

### Study cohort

COVID deviations and events were collected on all patients enrolled in Alliance trials through June 30, 2022. Enrollment trends were examined from January 1, 2019 (before the COVID-19 pandemic) through December 31, 2022. Patients enrolled by European Organization for Research and Treatment of Cancer (EORTC) on Alliance trials were not included. Analyses were done by pooling data across trials. No individual trials or patients were identified.

### Data collection

**Figure 1:**
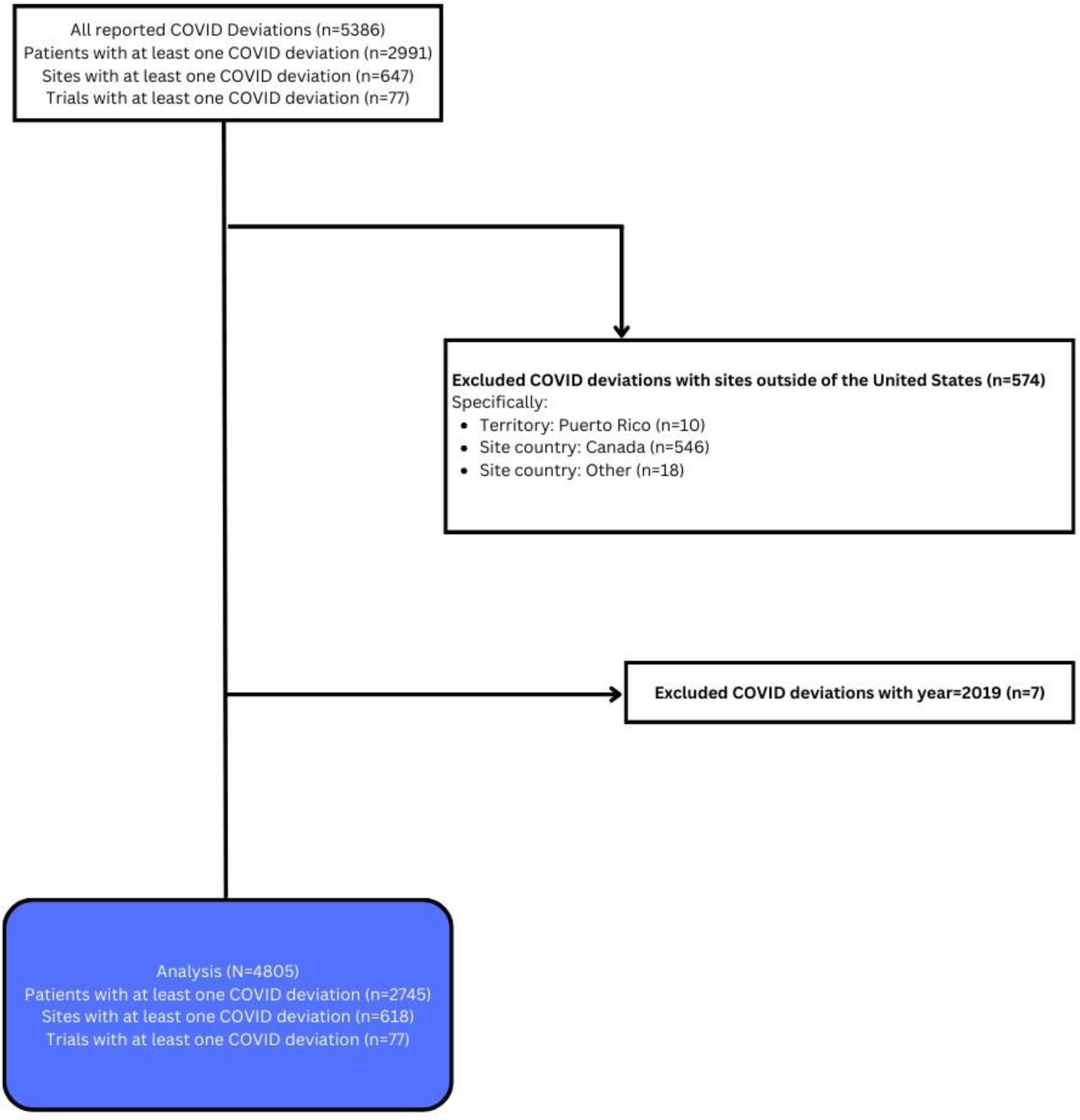
CONSORT diagram for COVID deviations^1^. **Note:** ^1^: COVID deviations that were categorized as “Other” were manually reviewed to identify the ones that should have been reported as a late procedure or as a virtual visit, or both. If multiple deviations of the same type were reported on the same day for the same patient, the duplicates were removed and only one of them was kept.

Figure 1, provides the CONSORT diagram of the deviation data collected during the pandemic. A centralized Medidata Rave database was created under a single Alliance protocol A152022 to capture a limited set of standard data related to COVID-19 testing and outcomes as well as all COVID-19 pandemic-related protocol deviations across all Alliance trials. The study was deemed exempt by the National Cancer Institute Central Institutional Review Board (NCICIRB) (dated 01/13/2021) because it was a pooled analysis and because no patient-identifying information was utilized. Data collection forms were designed to capture consistent data to allow the Alliance to evaluate impacts of the COVID-19 pandemic on Alliance trials and to meet both the NCICIRB and FDA basic reporting requirements. Sites were asked to report events within 14 days after notification of patient COVID-19 testing and/or pandemic-related deviations. COVID-19 reporting was required on all active Alliance trials that were either enrolling or conducting follow-up of trial patients. The central database was activated April 28, 2020, to collect COVID-19 related data at the start of the pandemic and remained open for data collection until June 30, 2022. Although all COVID testing was required to be collected in the initial rollout of the database, due to difficulties in capturing negative testing, negative tests were no longer required to be reported to the database beginning on December 15, 2020.

To improve data completeness, a computer algorithm was utilized to inspect all comments entered on key case report forms for words related to COVID-19 or the pandemic. When identified, if a COVID-19 event or protocol deviation had not yet been reported, the patient was flagged for further review by the data manager. If the comment was suspicious for a missed event or deviation, the site was queried for resolution.

### Outcome measures

The primary outcome measure was the number of patients enrolled on Alliance clinical trials. Additional covariates included in the analysis were age, gender, race, ethnicity, rural status, socioeconomic category, geographic region, and time categorized in 6-month intervals. Patient race and ethnicity were reported by the site at the time of study registration. Race and ethnicity were combined to form 4 groups: 1) White, Non-Hispanic or Latino; 2) Black or African American and Non-Hispanic or Latino; 3) Hispanic or Latino regardless of race; and 4) Other, which represents race or ethnicity that either was not reported or reported as unknown. Rural status, socioeconomic category, and geographic region were derived from 5-digit patient zip code. Rural/urban status was based on the county level of the patient’s zip code according to the 2013 National Center for Health Statistics Urban-Rural Classification Scheme for Counties. County-level Area Deprivation Index is calculated from surrogate indicators for income, employment, housing, and education associated with the 5-digit zip code for each patient; higher values correspond to increased socioeconomic disadvantage [8].

Secondary outcome measures included protocol deviations and specific deviation type, COVID-19 events defined as a positive or presumptive positive test for COVID-19, or premature treatment or study discontinuation due to COVID. Protocol deviations from sites outside the United States were excluded because they were not consistently reported [9].

Enrollment trends were summarized from January 1, 2019-December 31, 2022. COVID-19 deviations and events were summarized from January 1, 2020–June 30, 2022.

### Statistical analysis

The number and characteristics of patients enrolled on Alliance clinical trials and with a protocol deviation were evaluated using descriptive statistics. Differences in enrollment over time in the pandemic (pre-pandemic, early, mid, late), and by patient demographics were summarized descriptively using frequencies, percentages, and graphical techniques. Rates of protocol deviations and events determined to be due to COVID-19 were summarized over time. Data collection and statistical analyses were conducted by the Alliance Statistics and Data Management Center. Data quality was ensured by review of data by the Alliance Statistics and Data Management Center and by the study chairperson following Alliance policies. The COVID-19 study database was frozen on January 3, 2023.

## RESULTS

### Enrollment

**Figure 2:**
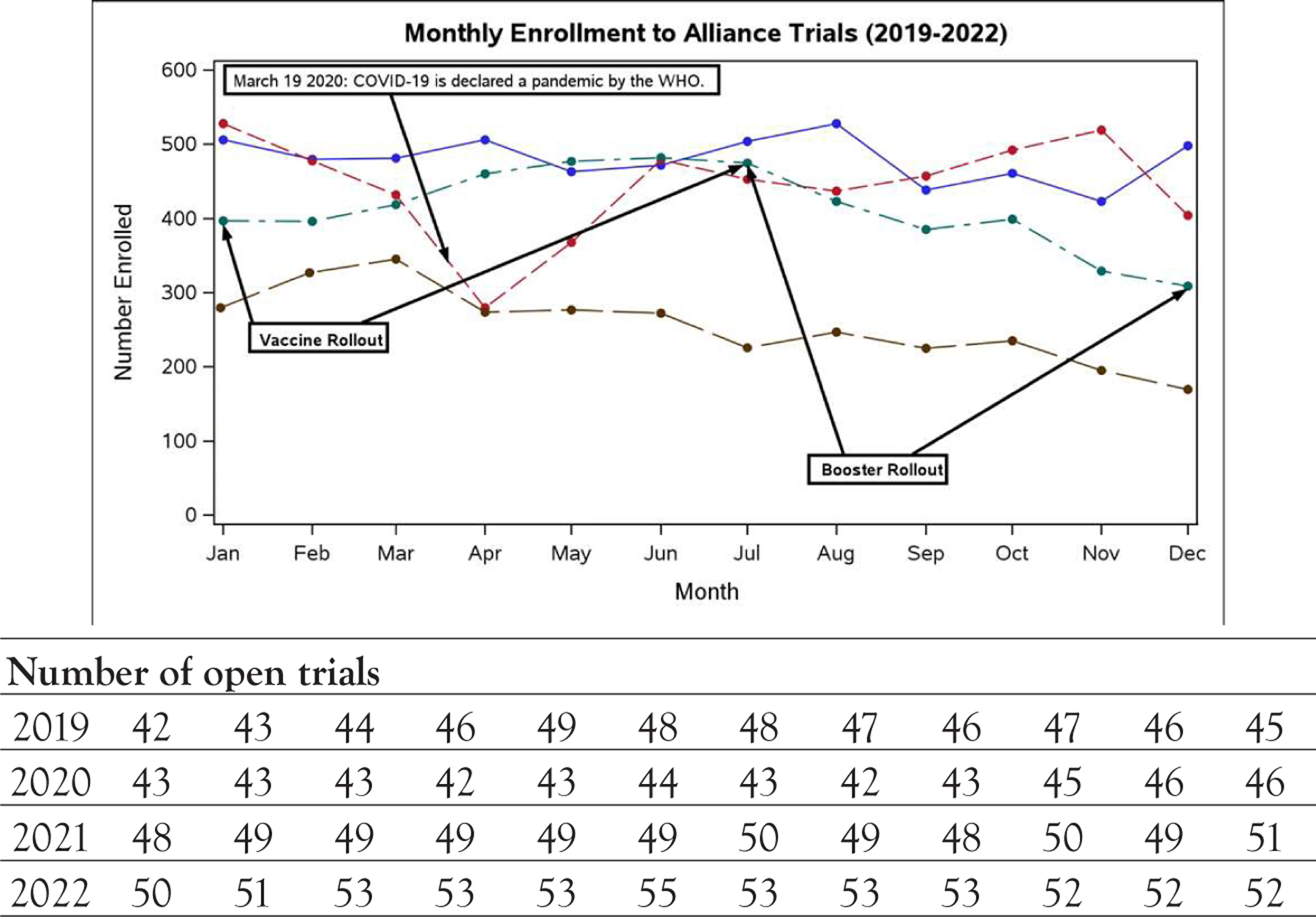
Alliance clinical trial enrollment (January 1, 2019-December 31, 2022). **Note:** 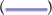 2019 (N=5760 Participants); 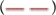 2020 (N=5327 Participants); 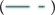 2021 (N=4951 Participants); 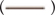 2022 (N=3073 Participants)

Enrollment trends were summarized from January 2019 to December 2022. The number of active Alliance clinical trials remained relatively constant from January 2019 to December 2022, ranging from 42 to 55 active trials per month (Figure 2). The number of patients enrolled decreased significantly during the COVID-19 pandemic, specifically from January to April of 2020 (Figure 2). The number of enrolled patients reached the pre-pandemic baseline by June of 2020. The total number of patients enrolled per year decreased over time, from 5760 patients in 2019 to 3073 patients in 2022, while the number of open trials was increased in 2022 compared to 2019. Two large, phase III trials (A011502, “Aspirin in Preventing Recurrence of Cancer in Patients with HER2 Negative Stage II-III Breast Cancer After Chemotherapy, Surgery, and/or Radiation Therapy;” and A011401, “Randomized Phase III Trial Evaluating the Role of Weight Loss in Adjuvant Treatment of Overweight and Obese Women with Early Breast Cancer”) completed enrollment and closed to further accrual on December 7, 2020 and February 15, 2021, respectively.

**Figure 3:**
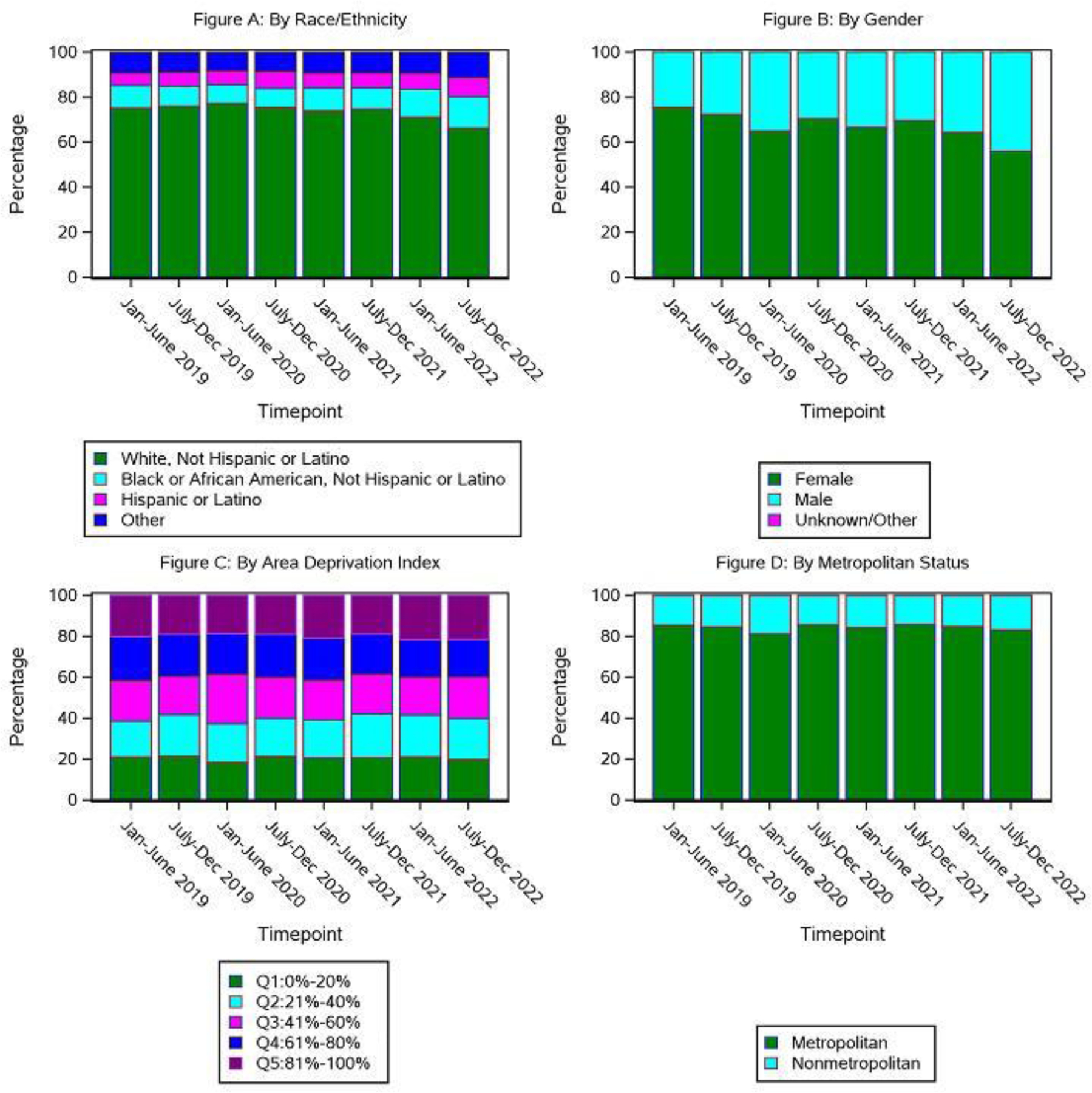
Accrual to alliance trials from January 1, 2019-December 31, 2022. **Note:** A) By race/Ethnicity; B) By gender; C) By area deprivation index; D) By metropolitan status.

**Figure 4:**
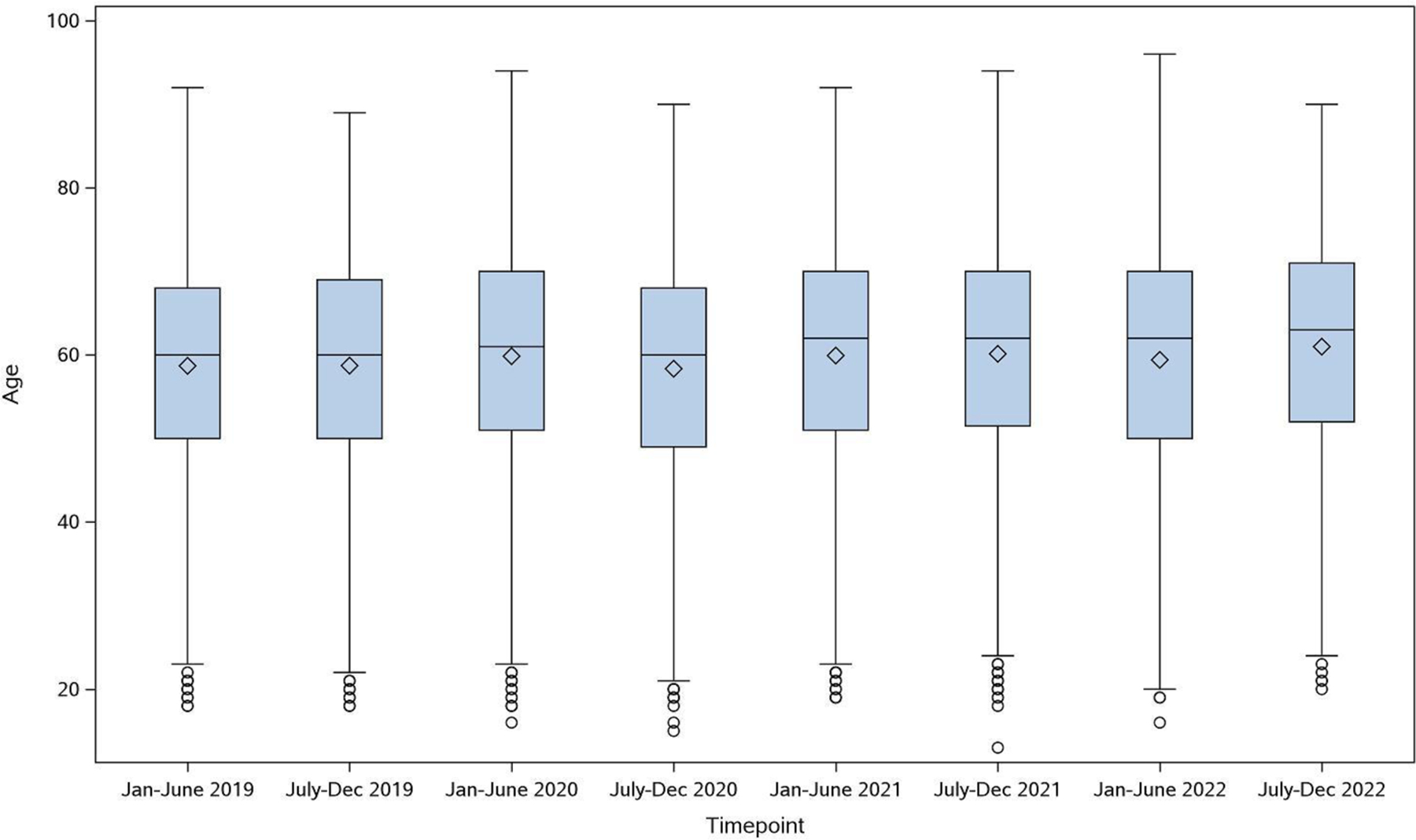
Distribution of accrual by age in years (median and interquartile range).

The racial demographics of enrolled patients did not vary significantly over the course of the pandemic (Figure 3A). Black patients comprised 10.1% of enrolled patients between January-June 2019, 8.4% in January-June 2020, 10.1% January-June 2021, and 12.5% January-June 2022. The proportion of Hispanic patients ranged from 5.5% to 8.6% between January 2019 and December 2022. A higher proportion of female compared to male patients were enrolled throughout the pandemic due to the number of active and enrolling breast cancer trials (Figure 3B). Median age of enrolled patients, and distribution of enrollment by metropolitan *versus* non-metropolitan and by area deprivation index remained consistent during this time (Figures 3C–3D and 4).

### Protocol deviations

All deviations reported from January 1, 2020–June 30, 2022, are summarized. A total of 4805 protocol deviations were reported for 2745 unique patients; of these 2065 were female (75%), 2223 White (81%), 283 Black (10%), and 226 Hispanic (8%) (Figure 1 and Supplemental Table 1). At least one protocol deviation was reported by 618 sites and 77 unique trials (Figure 1). Most deviations were reported for patients located in metropolitan areas (n=2391, 87%) with the highest proportion of patients reporting deviations located in the Midwest (n=915, 33%). Of the trials reporting protocol deviations, 47 (61%) were closed to accrual and 27 (35%) actively accruing. Trials reporting deviations included cancer control program studies (n=13, 17%), breast trials (n=11, 14%), genitourinary (n=9, 12%), lymphoma (n=8, 10%), and gastrointestinal (n=8, 10%) most commonly. The majority of reported protocol deviations involved use of a phone or virtual telemedicine visit (n=2167, 45%) or a late or missed study procedure (n=2150, 45%) (Table 1). The number of deviations decreased significantly over time, although the type of deviation remained relatively consistent (Supplemental Figure 1).

### COVID events

A total of 826 COVID events among 659 unique patients were reported among patients enrolled on an Alliance trial between January 1, 2020–June 30, 2022 (Supplemental Figure 2). The median age of patients with a COVID event was 60 years (range 13 to 88 years), 404 were female (61%), 50 Black (8%) and 544 White (83%), and 61 Hispanic (9%). Of these, 585 patients tested positive for COVID and 78 were presumptive positive. Reporting indicated that a total of 68 patients withdrew from treatment and 45 patients withdrew from the study altogether due to COVID, from an estimated total of 18,000 patients in follow-up during this timeframe (April 2020-June 2022).

## DISCUSSION

While enrollment patterns to cancer clinical trials have been summarized prior to the pandemic [10], this alliance COVID-19 pandemic study analyzed comprehensive data collected *via* the central COVID-19 case report forms across all active Alliance trials, along with data collected in the corresponding Alliance clinical trial databases. This pooled analysis allowed us to examine data on the prevalence and outcome of COVID-19 testing in oncology trial patients and the impact of the pandemic on oncology clinical trial accrual and conduct. A unique aspect of this effort is that this analysis included data for all oncology patients enrolled on all Alliance clinical trials during the pandemic, regardless of COVID-19 status. Thus, it provides valuable insight into the impact of the COVID-19 pandemic on NCI-sponsored cancer clinical trials.

Establishment of a centralized database enabled efficient and consistent collection of all COVID-19 events across all trials between April 2020-June 2022 that were either enrolling or conducting follow-up of study patients. While COVID-19 led to an immediate decline in enrollment across all patient populations, the number of enrolled patients reached the pre-pandemic baseline by June 2020. Age and racial demographics of enrolled patients did not change over the course of the pandemic, nor did the distribution of enrollment by area deprivation index. Despite concerns regarding timely and accurate protocol-specified clinical and safety assessments for cancer patients during the pandemic, our data suggest that the majority of reported protocol deviations were reported in a phone or virtual telemedicine visit, and both COVID-19 events and study discontinuations due to COVID-19 were rare in patients enrolled on Alliance clinical trials.

While our data is representative of only a single NCTN cooperative group, similar data has been reported, albeit retrospectively, in the Southwestern Oncology Group (SWOG). Investigators performed a retrospective cohort study to examine trends in SWOG enrollment over time, reporting that trial enrollment decreased significantly during the COVID-19 pandemic, especially among cancer control and prevention trials as compared to treatment trials [11]. Not surprisingly, enrollment declines were greater at study sites in states with the highest number of COVID-19 cases per 100,000 individuals [11].

Although overall enrollment has recovered, the COVID-19 pandemic has resulted in persistent changes in the conduct of National Clinical Trials Network (NCTN) cooperative group clinical trials, many of which will offer strategic advantages to administrative trial efficiency as well as improved patient access. The ASCO steering group on cancer care delivery and research in a post-pandemic environment recently published recommendations for modifying pre-pandemic policies and practices to improve patient access to high-quality cancer care and clinical trials [12]. These recommendations include: offering remote/virtual consent; allowing remote administration of treatment and conduct of patient assessments; and offering virtual options for site selection, study implementation, data collection, and protocol amendments, among others. Not only will these changes reduce the administrative burden to study sites, but these strategies will also reduce the financial burden to the patient (i.e. travel cost, time away from work, childcare or eldercare needs, etc.) and improve convenience of participation [12]. In a recent survey of clinical researchers who are members of ASCO, respondents indicate high levels of experience and comfort with telemedicine and remote patient monitoring, suggesting opportunities for further expansion of telemedicine in clinical trial procedures [13].

In these recommendations, ASCO also advised that the impact of changes made to studies during COVID-19 should be evaluated specifically regarding the diversity of patients participating in trials [12]. Over the course of the COVID-19 pandemic, Black and Hispanic individuals experienced increased rates of COVID-19 cases, hospitalizations, and death compared to White individuals [14,15]. Given the already existing disparity in clinical trial enrollment for individuals of Black race and Hispanic ethnicity, combined with higher rates of COVID-19 infection, an exacerbation in racial disparities in cancer clinical trial enrollment and subsequent health outcomes was projected. In a national survey of adults with cancer, Black and Hispanic respondents had greater odds of treatment delays, food insecurity, and concerns about financial stability and affordability of cancer treatment during the COVID-19 pandemic [16]. Fortunately, our data did not demonstrate a disproportionate impact of the COVID-19 pandemic on Alliance clinical trial enrollment rates, protocol deviations, or study withdrawal by patient race or ethnicity. Similarly, no difference was observed in patterns of decreased enrollment by race or ethnicity in SWOG clinical trials over the timeline of the COVID-19 pandemic [11]. However, the impact on participation rates across all NCTN trials and industry-sponsored trials is unknown.

When implementing changes to future study procedures, it will be important to consider potential unintended effects on minority patient populations. For example, recent data suggests that patients of Black race are less likely to have internet access or to use an online patient portal [17]. When implementing telehealth procedures for clinical trials, it will be critical to ensure that access to reliable internet and digital literacy do not preclude patients from trial participation. Additionally, it will be critical to ensure that remote informed consent processes maintain the same quality of communication as in-person visits and allow for adequate translation and/or differences in health literacy across populations. Changes in trial conduct emerging as a result of the COVID-19 pandemic may also result in other secondary effects as well. One study also demonstrated that enrollment in investigator-initiated therapeutic oncology trials during the pandemic was associated with decreased odds of reporting at least one serious adverse event (AE) [18]. Although serious adverse event reporting was not associated with the proportion of virtual visits in this single-center study, AE reporting may have been impacted by staffing shortages, protocol changes, or other administrative issues. Thus, it will be critical to anticipate and assess the impact of modifications in trial procedures on multiple outcomes in clinical trials in the future.

### Limitations

There are some limitations to this study. First, despite reporting requirements, it is difficult to ensure that all COVID events have been captured. We did utilize a systematic approach to identify missed reporting as described in the methods section, but we recognize that this was still a potential limitation. Second, COVID-19 testing practices changed during the pandemic, including testing location and documentation, which made it difficult for sites to capture data reliably. Third, since we did not capture negative COVID testing after December 2020, we could not evaluate the positivity rates. Despite these limitations, this study reports the results of a centralized, prospective, and comprehensive effort to summarize COVID-19 events, understand the impact of the pandemic on NCTN and NCORP clinical trial conduct, and inform future data collection efforts.

## CONCLUSION

A prospective, centralized COVID-19 database allowed for real-time tracking of the impact of the COVID-19 pandemic on NCTN Alliance cooperative group clinical trials. The pandemic resulted in immediate, significant declines in enrollment, which ultimately recovered to pre-pandemic levels. Differential changes in enrollment across patient populations were not observed. Common protocol deviations occurred as the result of telemedicine usage or late/missed study procedures, and few study withdrawals occurred due to COVID-19 infections. Post-pandemic changes to study procedures may offer increased flexibility and efficiency for trial sites and improved access to patients, which should be examined in future studies.

## Supplementary Material

Supplementary

## Figures and Tables

**Table 1:** Summary of protocol deviations (January 1, 2020-June 30 2022).

Deviation type, n (%)	Time points					
	Jan-June 2020 (N=2689)	July-Dec 2020 (N=1248)	Jan-June 2021 (N=510)	July-Dec 2021 (N=224)	Jan-June 2022 (N=134)	Total (N=4805)
**Eligibility**						
Disease criteria not met	1 (0.0%)	0 (0.0%)	0 (0.0%)	0 (0.0%)	0 (0.0%)	1 (0.0%)
Laboratory criteria not met	1 (0.0%)	1 (0.1%)	0 (0.0%)	0 (0.0%)	0 (0.0%)	2 (0.0%)
Other criteria not met	0 (0.0%)	2 (0.2%)	0 (0.0%)	0 (0.0%)	1 (0.7%)	3 (0.1%)
**Study intervention deviation**						
Significant deviation from planned dose (+/− 10%)	0 (0.0%)	0 (0.0%)	0 (0.0%)	1 (0.4%)	0 (0.0%)	1 (0.0%)
Significant deviation from planned dose (+/− 20%)	6 (0.2%)	0 (0.0%)	1 (0.2%)	0 (0.0%)	0 (0.0%)	7 (0.1%)
Discontinuation study drug non-protocol reason	8 (0.3%)	2 (0.2%)	1 (0.2%)	1 (0.4%)	0 (0.0%)	12 (0.2%)
Continuation of study drug should have discontinued	0 (0.0%)	0 (0.0%)	0 (0.0%)	1 (0.4%)	1 (0.7%)	2 (0.0%)
**Study visit**						
Late or missed study procedure	1292 (48.0%)	527 (42.2%)	184 (36.1%)	95 (42.4%)	52 (38.8%)	2150 (44.7%)
Late or missed QOL/PRO^*^	168 (6.2%)	83 (6.7%)	19 (3.7%)	11 (4.9%)	1 (0.7%)	282 (5.9%)
Phone or virtual visit	1125 (41.8%)	582 (46.6%)	287 (56.3%)	111 (49.6%)	62 (46.3%)	2167 (45.1%)
Other	36 (1.3%)	28 (2.2%)	11 (2.2%)	2 (0.9%)	14 (10.4%)	91 (1.9%)
**Study procedure**						
Efficacy assessment method different from baseline	1 (0.0%)	0 (0.0%)	1 (0.2%)	0 (0.0%)	0 (0.0%)	2 (0.0%)
Significant protocol deviation affecting safety	5 (0.2%)	0 (0.0%)	0 (0.0%)	0 (0.0%)	0 (0.0%)	5 (0.1%)
Other	23 (0.9%)	12 (1.0%)	2 (0.4%)	2 (0.9%)	1 (0.7%)	40 (0.8%)
**Serious GCP non-compliance**						
Study-specific procedures prior to informed consent	1 (0.0%)	0 (0.0%)	0 (0.0%)	0 (0.0%)	0 (0.0%)	1 (0.0%)
Informed consent not appropriately documented	5 (0.2%)	2 (0.2%)	1 (0.2%)	0 (0.0%)	0 (0.0%)	8 (0.2%)
Other	17 (0.6%)	9 (0.7%)	3 (0.6%)	0 (0.0%)	2 (1.5%)	31 (0.6%)

Note:

* : Quality of life/patient reported outcome.
