# Supplementary material for: Impact of the COVID-19 Pandemic on Cancer Clinical Trials: Alliance for Clinical Trials in Oncology Experience (Alliance A152022)
Source: J Clin Trials. Author manuscript; Available in PMC 2024 Sep 18. (PMC11410363)
Supplement: Supplementary [file NIHMS2013091-supplement-Supplementary.pdf]

Journal of Clinical Trials
OPEN ACCESS Freely available online

Research Article

Impact of the COVID-19 Pandemic on Cancer Clinical Trials: Alliance for Clinical Trials in Oncology Experience (Alliance A152022)

Rebecca A Snyder[1,2], Shauna L Hillman[3], Veronique Marcotte[3], Electra D Paskett[4], Suzanne George[5], Olwen Hahn[6], Sumithra J Mandrekar[3*]

[1]Department of Surgical Oncology, University of Texas MD Anderson Cancer Center, Houston, Texas, USA; [2]Department of Health Services Research, University of Texas MD Anderson Cancer Center, Houston, Texas, USA; [3]Department of Quantitative Health Sciences, Alliance Statistics and Data Management Center, Mayo Clinic, Rochester, Minnesota, USA; [4]Division of Cancer Prevention and Control, College of Medicine, The Ohio State University, Columbus, Ohio, USA; [5]Dana-Farber/Partners Cancer Care, Boston, Massachusetts, USA; [6]Alliance Protocol Operations Office, University of Chicago, Chicago, Illinois, USA

Supplemental Table 1: Characteristics of patients with a protocol deviation.

	Total (N=2745)
**Patient age**	
N	2745
Mean (SD) years	57.3(12.85)
Median years	58
Range years	13.0, 91.0
**Patient sex, n (%)**	
Female	2065(75.2%)
Male	680(24.8%)
**Patient race, n (%)**	
American Indian or Alaskan Native	12(0.4%)
Asian	91(3.3%)
Black or African American	283(10.3%)
More Than One Race	10(0.4%)
Native Hawaiian or Pacific Islander	6(0.2%)
Not Reported	60(2.2%)
Unknown	60(2.2%)
White	2223 (81.0%)
**Patient ethnicity, n (%)**	
Hispanic or Latino	226(8.2%)
Not Hispanic or Latino	2431(88.6%)
Not Reported	36(1.3%)
Unknown	52(1.9%)
**Patient race/ethnicity, n (%)**	
White, Not Hispanic or Latino	2028(73.9%)
Black or African American, Not Hispanic or Latino	274(10.0%)
Hispanic or Latino	226(8.2%)
Other	217(7.9%)
**Number of COVID-19 deviations**	
N	2745
Mean (SD)	1.8 (1.58)
Median	1
Range	1.0, 27.0

Note: Patients were included if they had one or more protocol deviations reported due to COVID -19 between January 1, 2020- June 30, 2022. Only patients from sites in the United States were included, except for Puerto Rico.

Correspondence to: Sumithra Mandrekar, Department of Quantitative Health Sciences, Alliance Statistics and Data Management Center, Mayo Clinic, Rochester, Minnesota, USA, E-mail: mandrekar.sumithra@mayo.edu

Received: 25-Jun-2024, Manuscript No. JCTR-24-32381; **Editor assigned:** 27-Jun-2024, PreQC No. JCTR-24-32381(PQ); **Reviewed:** 11-Jul-2024, QC No. JCTR-24-32381; **Revised:** 18-Jul-2024, Manuscript No. JCTR-24-32381(R); **Published:** 25-Jul-2024, DOI: 10.35248/2167-0870.24.14.573.

Citation: Snyder RA, Hillman SL, Marcotte V, Paskett ED, George S, Hahn O, et al (2024) Impact of the COVID-19 Pandemic on Cancer Clinical Trials: Alliance for Clinical Trials in Oncology Experience (Alliance A152022). J Clin Trials. 14:573.

Copyright: © 2024 Snyder RA, et al. This is an open-access article distributed under the terms of the Creative Commons Attribution License, which permits unrestricted use, distribution, and reproduction in any medium, provided the original author and source are credited.

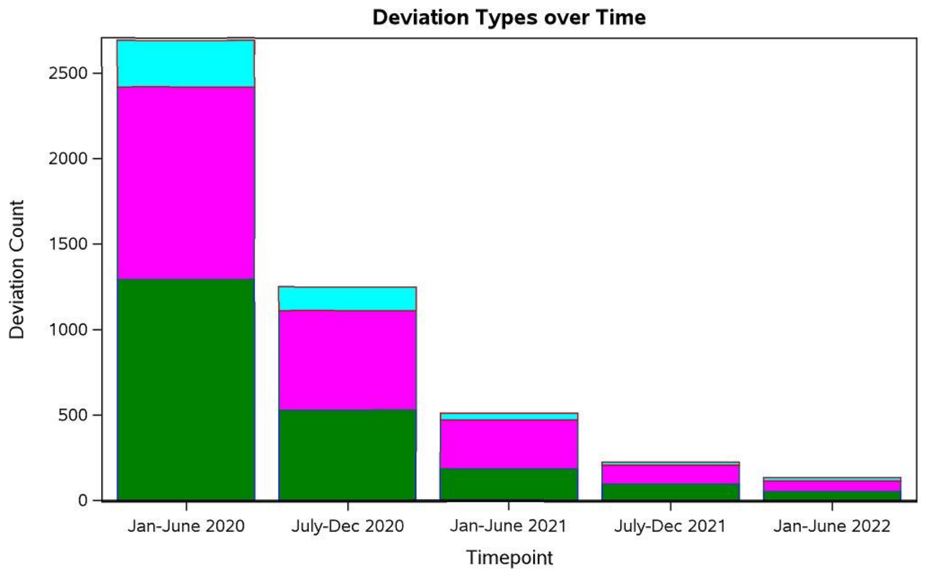

Supplemental Figure 1: Histogram of protocol deviations over study period (January 1, 2020-June 30 2022). **Note:** (■) Late or missed study procedure; (■) Phone or virtual/telemedicine visit; (■) Other deviation type.

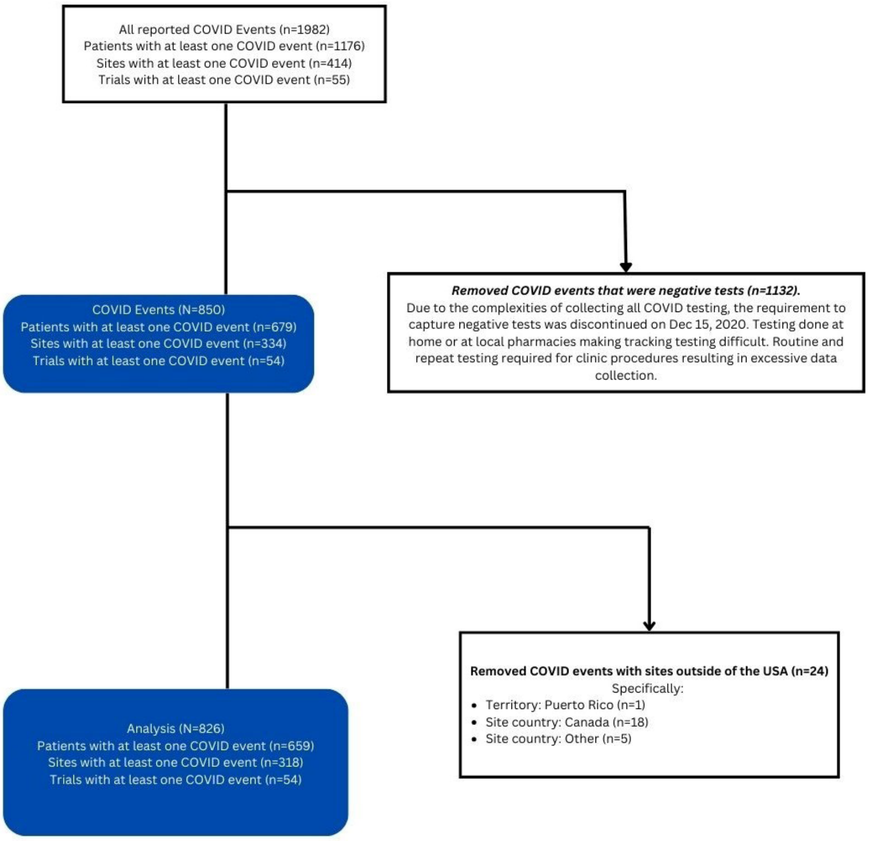

Supplemental Figure 2: CONSORT diagram for COVID events from January 2020-June 2022[1,2,3]. **Note: 1:** Participants categorized as tested positive include participants who tested positive and participants who were presumptive positive and tested positive within the same 7-day window; **2:** Participants categorized as presumptive positive include participants who were recorded as presumptive positive, but did not test positive within the same 7-day window; **3:** COVID events included all participants that were considered presumptive positive, tested positive or negative for COVID-19, went off treatment for any reason related to the COVID-19 pandemic or who withdrew from the parent trial due to any reason related to the COVID-19 pandemic. Both presumptive positive and testing positive events that occurred in the same 7-day window were counted as just 1 event.
